# The Incidence of Optic Cracks or Fractures During a Foldable AcrySof or Acriva BB Acrylic Monofocal Intraocular Lens Implantation via the Manual Monarch Injector System With the Cartridge in Phacoemulsification Surgery

**DOI:** 10.7759/cureus.38903

**Published:** 2023-05-11

**Authors:** Cem Evereklioglu, Yusuf Uysal, Hidayet Sener, Hatice Kübra Sönmez, Fatih Horozoglu

**Affiliations:** 1 Department of Ophthalmology, Erciyes University Medical Faculty, Kayseri, TUR; 2 Department of Ophthalmology, Gulhane Training and Research Hospital, Ankara, TUR

**Keywords:** tying forceps, monofocal, intraocular lens, glare, fracture, foldable, crack

## Abstract

Purpose: To evaluate the incidence of optic cracks and/or fractures during foldable acrylic intraocular lens (IOL) implantation via the manual Monarch delivery system with the cartridge and to determine factors that help to avoid such complications.

Methods: Small-incision phacoemulsification surgery was performed in 702 eyes with visually significant cataract formation. A foldable acrylic soft IOL (AcrySof^â^ MA60BM/MA30BA, Alcon, Fort Worth, TX, USA) or a single-piece acrylic soft IOL (Acriva BB^â^, VSY Biotechnology, Amsterdam, The Netherlands) was inserted in all eyes using a cartridge and viscoelastic agents (sodium hyaluronate, Healon^®^, Advanced Medical Optics, Santa Ana, CA, USA).

Results: Postoperative central, paracentral, or peripheral optic cracks or fractures were encountered in a total of six of 702 eyes (0.85%). Four of six lenses (0.57%) had optic cracks within the IOL substance, whereas two of 702 cases (0.28%) had full-thickness IOL fractures in the substance in multiple locations. Three of the four lenses with optic cracks were noted to be handled by tying forceps during the cartridge insertion, and one of them was the complication of holding forceps. Two IOLs with full-thickness optic fractures were encountered during the insertion of the IOLs in the capsular bag as a result of direct trauma to the lens optic by the plunger of the injector system overriding the lens optic during cartridge passage. None of the patients suffered from glare or other visual disturbances postoperatively, and, therefore, none of the six eyes required lens replacement.

Conclusion: The unintentional extensive pressure effect of the forceps during the holding process of the IOL or the direct trauma to the lens optic by the plunger of injector systems may cause optic cracks or fractures. Physicians should continue to monitor the eyes postoperatively regularly and must determine the benefits and risks to be derived from lens replacement, if such patients complain of significant glare, image degradation, and visual disturbances. We recommend the use of preloaded lenses, which have their own delivery systems and cartridges, to minimize the risk of such complications.

## Introduction

Techniques for cataract surgery demonstrate rapid development and evolution, and self-sealing, small-incision cataract surgery with phacoemulsification has become the gold standard, which led to increased implantation of foldable intraocular lenses (IOLs) [1,2]. Acrylic foldable IOLs were developed for small-incision cataract surgery, and the incidence of complications has significantly decreased with the development of new foldable IOLs of various materials and shapes, which is the subject of continuing research [3-5].

Although a forceps is generally preferred to insert an acrylic IOL when preloaded lenses are not available, it may bring bacteria into the eye. Therefore, using an injector with a cartridge or preloaded system has some advantages to inserting acrylic IOLs because the IOL does not contact the lid or conjunctiva preoperatively [6]. The clinical studies of the AcrySofâ or Acriva BBâ posterior chamber IOLs began decades ago and were released on the market, which introduced considerable technical innovations, including a high refractive index, square edges, and slow, controlled unfolding resulting from its plasticity [7,8]. These lenses were conducted with the lens being intended for implantation in the capsular bag with a safe and effective visual correction of aphakia.

Surface deposits were observed on the posterior IOL surface that was injected through a cartridge filled with sodium hyaluronate [9,10]. Herein, we report the incidence of optic cracks or fractures of soft foldable IOLs after phacoemulsification surgery and suggest some tricks to avoid such unwanted IOL damages during the IOL insertion into the cartridge and intraocular capsular bag implantation.

## Materials and methods

This retrospective study was performed in the Department of Ophthalmology, Division of Cataract and Refractive Surgery, Gulhane Training and Research Hospital and Erciyes University Medical Faculty, and Mayaeye Hospital, Kayseri, Türkiye. The investigation conformed to the tenets of the Declaration of Helsinki and was approved by the Institutional Review Board (Approval number: 2023/234).

We evaluated 702 eyes and reported the presence of postoperative central, paracentral, or peripheral optic cracks or full-thickness optic fractures during capsular bag implantation of one soft foldable acrylic lens following normal phacoemulsification with a no-stitch clear corneal incision. An AcrySofâ (Alcon, Fort Worth, TX, USA) MA30BA/MA60BM or Acriva BBâ (VSY Biotechnology, Amsterdam, The Netherlands) acrylic foldable IOL was entirely inserted in the cartridge with sufficient lubrication of the optic with a viscoelastic material (Healon®, Advanced Medical Optics, Santa Ana, CA, USA), and the IOL was finally implanted in the capsular bag using an injector.

After complete preoperative ocular examinations were performed, unilateral phacoemulsification surgery with one of the abovementioned soft foldable posterior chamber IOL was implanted. Each eye was accepted as a separate case. All surgeries were performed by the same standardized approach and were examined by experienced ophthalmologists or residents in ophthalmology under the supervision of an experienced skilled surgeon. The pupils were dilated with topical cyclopentolate 0.5% about half an hour before the surgery. Two paracenteses were performed on the temporal and nasal corneal periphery. Viscoelastic material was injected into the anterior chamber (Healon, Advanced Medical Optics, Santa Ana, CA, USA), and a 2.8 mm clear corneal incision was made with a phaco knife superiorly. An anterior continuous curvilinear capsulorhexis was manually performed with a needle that was completed by routine capsulorhexis forceps. After hydrodissection was completed, hydrodelamination in cases with nuclear sclerosis was performed. Soft cataract cases with posterior capsule opacification did not require hydrodelamination. The nucleus was emulsified in the posterior chamber and the remaining lenticular material was aspirated by the phaco probe. After the meticulous cortical cleanup was performed, the anterior chamber and the capsular bag were filled with viscoelastic material again, and a foldable acrylic IOL was implanted in the bag and inserted through the main superior surgical opening [11]. After the folding process of the soft IOL within the cartridges was completed, the insertion of the soft acrylic posterior chamber IOLs was performed via the manual Monarch delivery system (Alcon, Fort Worth, TX, USA). The tip of the cartridge was inserted through the main superior incision up to the anterior chamber and then the plunger was advanced similarly to insert the foldable IOL. When the entire optic and leading haptic have been inserted in the bag position, the cartridge was withdrawn, and the remaining haptic was inserted in the capsular bag. During this stage, optic cracks or fractures, if present, were noted preoperatively in some cases while the lenses were in the capsular bag position. We did not attempt to replace the IOLs with cracks because this could be more traumatic to the eye, and, therefore, the risk of exchange seemed to be greater than the benefit. Similarly, we did not remove the fractured IOLs because the full-thickness fractures were peripherally located, which were under the upper eyelid, and, therefore, did not affect the visual acuity. Viscoelastic material was removed from the eye by irrigation-aspiration probes, and the surgery was ended with the injection of an intracameral antibiotic. Postoperatively, the eyes received topical ketorolac 0.5% ophthalmic solution four times a day for four weeks, topical dexamethasone 0.1% drops six times a day for the first week, and four times a day for the remaining three weeks. Similarly, both groups received antibiotic drops four to six times a day for a month. Follow-up examinations were repeated on the postoperative first, third, and seventh days of the first month and at six-month intervals thereafter.

## Results

**Figure 1:**
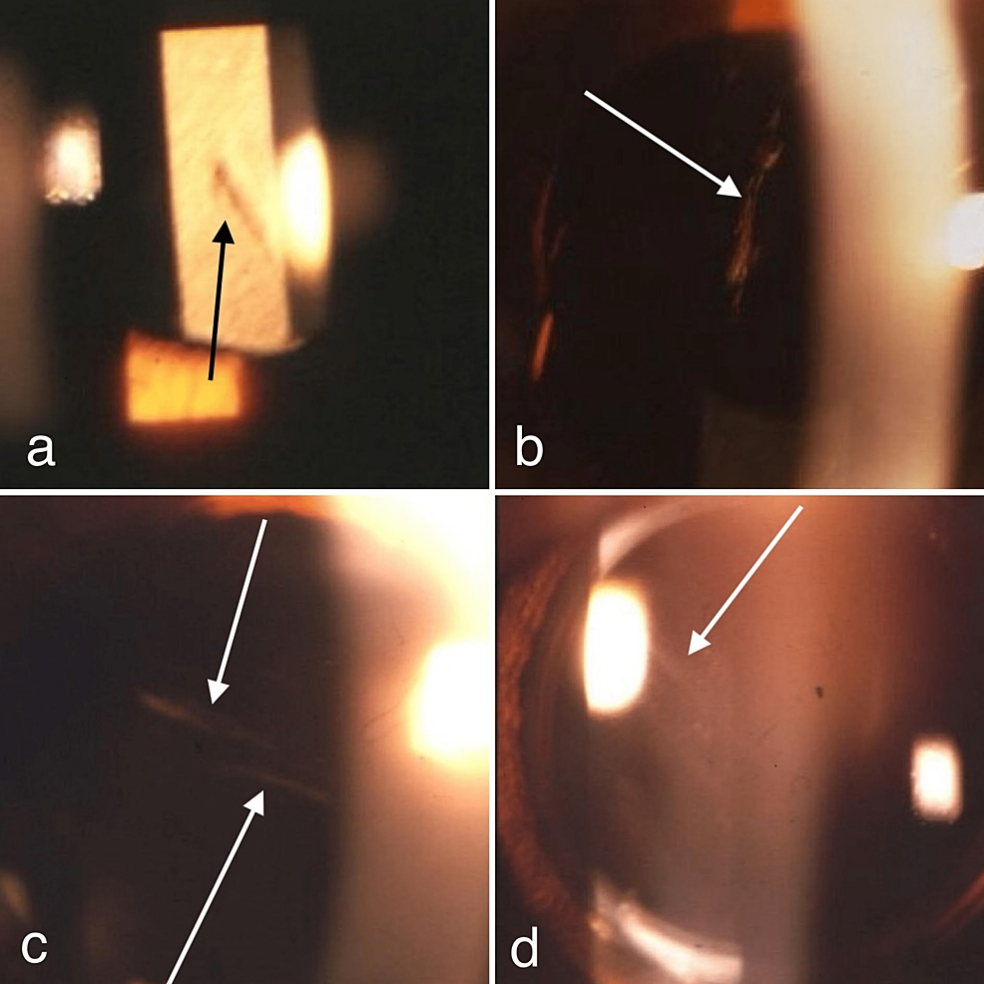
Postoperative biomicroscopic photographs of four implanted in-the-bag intraocular lenses demonstrate optic cracks on the lens surfaces. a. A central optic crack in the oblique direction by retro-illumination (arrow). b. A central optic crack in the vertical direction on slit-lamp biomicroscopy (arrow). c. Paracentral double optic cracks in the oblique direction on slit-lamp biomicroscopy (arrows). d. Paracentral optic crack in the oblique direction on slit-lamp biomicroscopy (arrow).

We have used this procedure to perform uneventful IOL implantation in 702 eyes and evaluated all IOLs in detail at early per- or postoperative days. Routine slit-lamp biomicroscopy revealed that four eyes (0.57%) had central (Figures 1a, 1b) or paracentral (Figures 1c, 1d) optic cracks among 702 operated eyes.

These four lenses were either acrylic MA60BM or MA30BA soft foldable IOLs (Alcon, Fort Worth, TX, USA). We realized that three of these four lenses had been handled with routine tying forceps and one with routine holding forceps. On the other hand, although the cracks in two of four cases were in the center of the lenses, none of the patients suffered from glare, image degradation, or other visual disturbances. Therefore, none of the eyes required lens replacement with a new lens. In addition, all eyes were followed up for at least six months, and there was no crack-related change in lens materials, and none of the lenses showed any surface accumulation or precipitation of any kind by slit-lamp biomicroscopy, suggesting that such cracks were in the substance of the lens optic, not on the surface.

**Figure 2:**
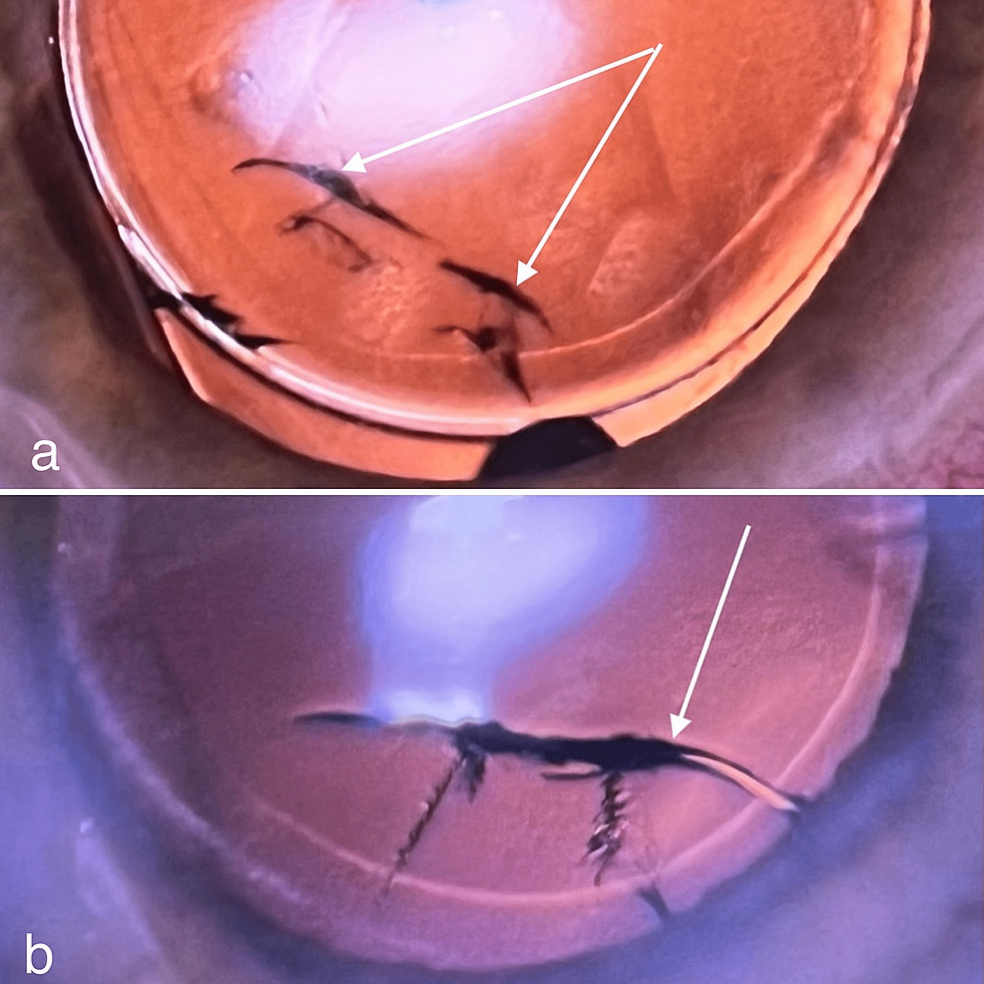
Perioperative photographs of two implanted in-the-bag intraocular lenses demonstrating full-thickness optic fractures. a. Peripheral optic fractures at four points by retro-illumination (arrows). b. Peripheral optic fractures in π-shape demonstrated by retro-illumination (arrow).

In cases with Acriva BBâ foldable acrylic IOL implantation (VSY Biotechnology, Amsterdam, The Netherlands), two lenses (0.28%) demonstrated full-thickness fractures at the superior aspect of the optics preoperatively at four different points (Figure 2a) or in a single “π-shape” fracture demonstrated by retro-illumination (Figure 2b). The surgeons were experienced in both cases with optic fractures.

These full-thickness optic fractures were encountered during the insertion of the IOLs in the capsular bag as a result of direct lens trauma from the plunger of the delivery system overriding the lens optic during their cartridge passages. Similar to optic cracks, none of the patients with optic fractures suffered from glare, image degradation, or other visual disturbances postoperatively and none of the cases needed IOL replacement surgery. Taken together, a total of six out of 702 cases had optic cracks or fractures (0.85%).

## Discussion

Soft acrylic IOLs were folded very efficiently, and a small number of viscoelastic agents, which were routinely used at both surfaces of the lenses in all of our cases, facilitated the IOL unfolding in the eye and, therefore, eased the process of controlled lens insertion and positioning. However, cracked or fractured IOLs can be left in place because of their excellent biocompatibility, especially if they are at the periphery of the lens optic. In cases with central or paracentral fractures, the lenses should be preoperatively replaced by undamaged ones. The visual results after cataract surgery can be affected by the surface or substance properties of an IOL [12], and high-quality properties are essential for the safety of an IOL. In the present research, optic cracks or complete fractures were seen on some lenses, which was related to the use of unsuitable handling of the lenses from their central or paracentral optic part or careless plunger advancement to insert the foldable IOL.

The folding and insertion processes of soft IOLs into the cartridges impose considerable stress on the lens optics due to strong compression forces during the handling and ejecting stages [13]. Therefore, it is imperative for IOLs to be made of high optical quality so that they demonstrate exceptional flexibility and durability to prevent permanent surface or substance deformations. Indeed, Singh et al. [14] demonstrated that cartridge cracks might develop in 52 of 350 eyes that used Healon to load the IOL. In addition, there was evidence of the plunger trauma overriding the optic in each case of a cracked cartridge. In our large series, there was evidence of the plunger overriding the IOL optic but without a cartridge crack in two cases. The excessive pressure effect of the forceps during the holding process might have resulted in our four cases with optic cracks, though one of the present lenses was handled with holding forceps. Thus, the cataract surgeon should confirm that the IOL is not handled by its optic and is lubricated by a satisfactory amount of viscoelastic so that it can be smoothly and easily inserted into the cartridge. Although all our six patients had no visual complaints of any kind, it is still possible to crack or fracture the lens significantly, and some cracks or fractures may result in glare or other visual disturbances under certain lighting conditions. In addition, the long-term effects of such lenses with optic cracks/fractures cannot be estimated, although they seem to have excellent biocompatibility.

We also observed some minor peripheral surface scrapes during IOL manipulation during the insertion process in the cartridge. But still, the implantation of IOLs using injectors has been found to be safer than with forceps [15]. It is clear that handling and inserting maneuvers result in optic cracks due to the incorrect use of the forceps, which cause micro-damages on the substance of the IOL and are dependent on the capability of the physician. Similarly, the plunger of the delivery (injector) systems may be harmful to the IOL optics if the cataract surgeon is not attentive during the insertion of the lens within the cartridge. The preloaded IOLs, in turn, are easily taken from their original packages, which can be injected into the capsular bag safely. Therefore, our suggestions to avoid such damages are as follows; (1) the soft acrylic IOLs as well as other kinds of foldable lenses should not be grasped from the optical area at all; (2) bfore the actual folding process, the lens should be handled by the haptic portion only; (3) the lens should be rinsed thoroughly using irrigation solutions; (4) all instrumentations should scrupulously be cleaned to minimize the occurrence of marks on the lens due to folding; (5) the lens should be folded with an appropriate folding system or equivalent forceps with round edges and smooth surfaces; (6) sufficient amount of viscoelastics should be placed at the back and front surfaces of the lens prior to folding process; (7) the lens should carefully be examined before its insertion to ensure that particles have not adhered during handling, and finally; (8) the plunger of the injector system should meticulously be followed during its passage within the cartridge; (9) pre-loaded IOL injectors with single piece foldable lenses should be preferred, if available.

There are two limitations to the study. First, it is a retrospective study, and therefore, prospective evaluations of various IOL materials from other manufacturers may reveal different results. Second, the cataract operations were performed by both experienced surgeons and residents in the department of ophthalmology clinics. So, single-surgeon insertion and implantation of foldable IOLs using different kinds of cartridges and holding/folding forceps may reveal different incidences of cracks or fractures after uneventful phacoemulsification surgery.

## Conclusions

Because various IOL injector systems exist in many manufacturers, the surgeon should be competent and know the difference between the preloaded lenses and manually handled delivery systems, all of which have their own injector system and cartridges. Indeed, pre-loaded IOL injectors seem to be becoming more common all around the world. Although we use more and more IOL cartridges and then load the soft lenses into the related devices, three-piece lenses have also been used in some centers, and physicians should continue to monitor patients postoperatively regularly basis and must determine the benefits and risks to be derived from lens replacement if such patients complain troublesome glare and optic or visual disturbances.
